# The association of plasma TMAO and body composition with the occurrence of PEW in maintenance hemodialysis patients

**DOI:** 10.1080/0886022X.2025.2481202

**Published:** 2025-03-20

**Authors:** Xinran Wang, Xinyue Peng, Jun Liu, Shiqi Tang, Xinyu Yang, Jianwen Wang

**Affiliations:** a Department of Nephrology, The Third Xiangya Hospital, Central South University, Changsha, China; b The Critical Kidney Disease Research Center of Central South University, Changsha, China; c Department of Nephrology, The First Affiliated Hospital of Hunan University of Medicine, Huaihua, China

**Keywords:** Trimethylamine N-oxide, body composition, protein-energy wasting, maintenance hemodialysis, chronic kidney disease

## Abstract

**Introduction:**

This study aims to explore the relationship between trimethylamine N-oxide (TMAO), body composition, and protein-energy wasting (PEW) in patients undergoing maintenance hemodialysis (MHD).

**Methods:**

A total of 127 MHD patients participated in this study. Body composition was measured using the InBody770 multi-frequency body composition analyzer. Plasma TMAO concentrations were assessed by ELISA. Cross-sectional analysis was performed after collecting demographic data, dialysis-related data, laboratory parameters, and body composition data from MHD patients.

**Results:**

In MHD patients, the PEW group exhibited lower levels of hemoglobin (Hb), albumin (ALB), transferrin (TF), creatinine (Cr), triglycerides (TG), prealbumin (PALB), soft lean mass (SLM), body mass index (BMI), percent of body fat (PBF), arm muscle circumference (AMC), and phase angle (PHA) compared to the non-PEW group, while C-reactive protein (CRP) and trimethylamine-N-oxide (TMAO) levels, as well as Extracellular Water/Total Body Water (ECW/TBW) ratio, were higher in the PEW group than in the non-PEW group. After full adjustment, TMAO and ECW/TBW ratio were independent risk factors for PEW in MHD patients. Further, plasma TMAO levels correlated negatively with Cr, ALB, Hb, BMI, and PHA, and positively with ECW/TBW in MHD patients with PEW. The ROC curve analysis indicated that the area under the curve (AUC) for plasma TMAO in predicting PEW in MHD patients was 0.788.

**Conclusions:**

Plasma TMAO levels and certain body composition are associated with the occurrence of PEW in MHD patients. Plasma TMAO levels appear to serve as a potential predictive marker for the onset of PEW.

## GRAPHICAL ABSTRACT

**Figure:**
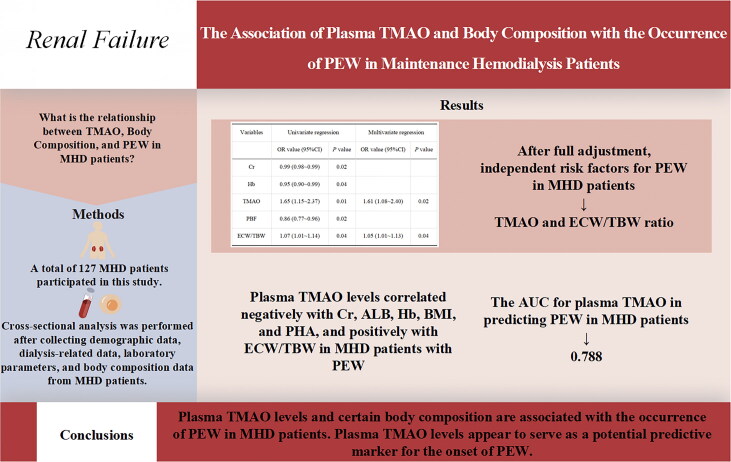

## 1. Introduction

Protein-energy wasting (PEW) is a common complication in patients with chronic kidney disease (CKD). The incidence of PEW increases with the decline in glomerular filtration rate (GFR), being highest in patients with end-stage renal disease (ESRD) [1]. Moreover, PEW is even more prevalent among patients undergoing maintenance hemodialysis (MHD) [1,2]. PEW is defined as the loss of somatic and circulating body protein and energy reserves, characterized primarily by a reduction in the body’s protein and energy stores [3,4]. Based on the definition and characteristics of PEW, we speculate that body composition in MHD patients may be associated with PEW. The occurrence of PEW is closely associated with poor outcomes in patients with CKD, significantly impairing their quality of life, particularly in those undergoing MHD [5,6]. Research has demonstrated that PEW in MHD patients is linked to impaired quality of life and increased rates of cardiovascular mortality, infection-related mortality, and overall mortality [1]. Therefore, the prevention and treatment of PEW are crucial for patients with end-stage kidney disease undergoing MHD.

Trimethylamine N-oxide (TMAO) is a metabolic product derived from dietary substances such as choline and L-carnitine. Choline and L-carnitine that are not absorbed in the small intestine are metabolized by gut microbiota into trimethylamine (TMA) in the colon. TMA is then oxidized to TMAO in the liver by flavin-containing monooxygenase 3 (FMO3) [7,8]. TMAO is a free, water-soluble, low-molecular-weight, gut-derived microtoxin, with a molecular weight of 75.1 g/mol [9]. TMAO is cleared by the kidneys through a combination of glomerular filtration and tubular secretion [10]. Under normal physiological conditions, circulating TMAO is almost completely excreted *via* urine, resulting in its rapid clearance [11]. In patients with CKD, plasma TMAO concentrations significantly increase as renal function declines [12,13]. TMAO can be cleared by hemodialysis, and its clearance rate is comparable to that of creatinine [14]. The toxic mechanisms of TMAO are associated with the induction of renal fibrosis and the enhancement of immune responses in atherosclerosis [9]. In addition, TMAO levels are closely linked to a variety of diseases, including cardiovascular and cerebrovascular conditions, type 2 diabetes mellitus (DM), hypertension, renal insufficiency, and cancer, among others [12]. Cardiovascular disease (CVD) is the primary cause of death in patients with CKD, and the risk of mortality from CVD is even greater in individuals with ESRD [15].Elevated levels of circulating TMAO are associated with an increased risk of CVD and mortality in patients with CKD, affecting their overall prognosis [16]. Research has indicated that inhibiting TMAO production by gut microbiota can ameliorate renal damage caused by chronic nephritis and significantly downregulate various markers of tubulointerstitial fibrosis [17]. PEW is closely associated with gut microbiota dysbiosis in patients undergoing hemodialysis [18]. Therefore, we hypothesize that there is a correlation between the gut microbiota metabolite TMAO, body composition, and PEW.

To gain a deeper understanding of the factors influencing PEW in MHD patients, this study conducted a cross-sectional clinical investigation of ESRD patients who were undergoing MHD. The investigation included collecting demographic and dialysis-related data, laboratory parameters and body composition. This study focuses on evaluating several important factors that influence circulating TMAO levels and the relationship between TMAO and body composition. The aim was to explore the clinical characteristics and influencing factors of PEW, provide a basis for clinical assessment of PEW in MHD patients, and establish a foundation for effective prevention and treatment of PEW.

## 2. Methods

### 2.1. Study population

This cross-sectional study enrolled 127 ESRD patients aged 18 to 80 years from the Department of Nephrology at the Third Xiangya Hospital, Central South University, who had been undergoing regular hemodialysis for more than 3 months. Exclusion criteria included prior history of peritoneal dialysis, kidney transplantation, autoimmune diseases, cancer, chronic liver disease, infections, gastrointestinal disorders, enteral or parenteral nutrition, special diets (such as consumption of red meat, seafood, egg yolks ≥ 5 times/week, as well as vegetarian or exclusively meat-based diets), and recent use (within the past month) of medications such as sevelamer, probiotics, antibiotics, vitamins, non-steroidal anti-inflammatory drugs, metformin, steroids or immunosuppressive agents. Additionally, individuals with metallic medical implants, pacemakers, amputations precluding body composition analysis were excluded. The type of hemodialysis technique, including medium-flow or high-flow hemodialysis and hemodiafiltration (HDF), was chosen based on the dialysis adequacy for each patient, with a dialysis frequency of three times per week, each session lasting 4 h. Ethical approval for this study was obtained from the Ethics Committee of the Third Xiangya Hospital, in accordance with the principles of the Declaration of Helsinki (Approval No. 23336). All participants underwent interviews and provided signed informed consent forms.

### 2.2. Data collection

Demographic and dialysis-related data were collected, including age, gender, duration of dialysis and urea clearance index (Kt/V). Fasting venous blood samples were collected at least 36 h after a hemodialysis session (prior to the next hemodialysis session), to assess various parameters: white blood cell (WBC), platelet (PLT), C-reactive protein (CRP), hemoglobin (Hb), albumin (ALB), ferritin (Ferr), transferrin (TF), serum iron (SI), blood urea nitrogen (BUN), creatinine (Cr), parathyroid hormone (PTH), high-density lipoprotein cholesterol (HDL-C), low-density lipoprotein cholesterol (LDL-C), triglycerides (TG), total cholesterol (TC), prealbumin (PALB), endogenous creatinine clearance rate (Ccr), and TMAO. TMAO levels were assessed using ELISA kits (mlbio, Shanghai, China).

### 2.3. Body composition

Using the InBody770 multi-frequency body composition analyzer (InBody, KOR) based on BIA principles, body composition measurements were conducted under the guidance of a specialized nursing team. The body composition analysis was performed on MHD patients at least 36 h after a hemodialysis session (prior to the next hemodialysis session), with the analysis completed on the same day as blood sample collection. Parameters collected included: body mass index (BMI), which measures the degree of adiposity; soft lean mass (SLM), representing the quality of lean tissues such as muscle in the body; skeletal muscle index (SMI), assessing skeletal muscle content; percent of body fat (PBF), indicating the percentage of total body weight composed of fat tissue; arm muscle circumference (AMC); extracellular water/total body water (ECW/TBW), the ratio of extracellular fluid volume to total body water, reflecting body volume load; and phase angle (PHA), the ratio of human impedance to reactance, reflecting cellular integrity and quality.

### 2.4. Diagnosis of PEW

The diagnosis of PEW was made according to the criteria set by the International Society of Renal Nutrition and Metabolism (ISRNM) [19]: (1) Serum Biochemical Indicators: ALB <38 g/L, PALB <0.3 g/L, or TC < 2.59 mmol/L; (2) Body Mass: BMI <22 kg/m^2^ (or <23 kg/m^2^ in individuals >65 years), >5% weight loss within 3 months, >10% weight loss within 6 months, or body fat <10%; (3) Muscle Mass: >10% reduction in mid-arm muscle circumference over 6 months; and (4) Dietary Intake: inadequate intake of protein or energy for at least 2 months (protein intake <0.8 g/kg/day in dialysis patients, or energy intake <25 kcal/kg/day) without intentional restriction. PEW is diagnosed when at least three of these four categories are met, with at least one test result meeting the criteria in each category.

### 2.5. Statistical methods

Data were analyzed using SPSS version 23.0 software. Firstly, the normality of the data was assessed using the Kolmogorov-Smirnov (K-S) test. For normally distributed continuous variables, results are presented as mean ± standard deviation. For non-normally distributed data, results are presented as median (P25, P75). Categorical variables are presented as n (%). Group comparisons were conducted using Student’s t-test for normally distributed data and non-parametric tests (Mann-Whitney U test) for non-normally distributed data, while categorical variables were analyzed using the chi-square test. Pearson correlation analysis was used for variables with normal distribution and Spearman correlation for non-normally distributed variables. Logistic regression analysis was performed to explore factors influencing PEW. Receiver operating characteristic (ROC) curve analysis was conducted to evaluate the predictive ability of plasma TMAO levels for PEW in dialysis patients. A significance level of *p* < 0.05 was considered statistically significant.

## 3. Results

### 3.1. Comparison of demographic, dialysis-related data and laboratory parameters between PEW and non-PEW groups in MHD patients

A total of 127 ESRD patients who were undergoing MHD were included in this study. Using the ISRNM clinical diagnostic criteria [19], patients were stratified into PEW and non-PEW groups. There were 73 patients in the PEW group and 54 in the non-PEW group, resulting in a prevalence of 57.48%. As shown in Table 1, in MHD patients, the PEW group exhibited lower levels of Hb, ALB, TF, Cr, TG, and PALB compared to the non-PEW group, while CRP and TMAO levels were higher in the PEW group than in the non-PEW group. These differences were statistically significant (*p* < 0.05). However, there were no statistically significant differences between the two groups in age, sex, duration of dialysis, Kt/V, WBC, PLT, Ferr, SI, BUN, PTH, LDL-C, HDL-C, TC, and Ccr.

**Table 1.** Comparison of demographic, dialysis-related data and laboratory parameters between MHD patients in PEW and non-PEW groups.

Variables	PEW group	non-PEW group	*P* value
	*N* = 73	*N* = 54	
Age (years)	50.52 ± 13.21	54.04 ± 15.11	0.17
Male	42 (57.53)	35 (64.81)	0.40
Duration of dialysis (months)	16.00 (11.00–49.00)	14.50 (9.75–35.25)	0.27
Kt/V	1.33 ± 0.11	1.34 ± 0.10	0.53
WBC (10^9^/L)	6.21 ± 1.87	6.80 ± 2.74	0.15
Platelet (10^9^/L)	175.12 ± 74.36	193.69 ± 77.78	0.17
C-reactive protein (mg/L)	10.61 (5.00–18.30)	5.51 (2.47–21.6)	0.03
Hemoglobin (g/L)	85.52 ± 18.55	93.59 ± 21.63	0.02
Albumin (g/L)	31.16 ± 4.22	38.06 ± 3.58	< 0.01
Ferritin (ng/mL)	185.65 (73.75–485.83)	159.5 (90.73–419.43)	0.67
Transferrin (g/L)	1.66 ± 0.57	1.88 ± 0.55	0.03
Serum iron (μmol/L)	10.4 (7.2–18.3)	9.7 (6.9–15)	0.38
BUN (mmol/L)	23.19 ± 10.47	24.45 ± 10.15	0.50
Creatinine (μmol/L)	753.30 ± 84.41	1053.49 ± 71.01	< 0.01
Parathyroid hormone (pg/mL)	246.07 (91.70–449.06)	238.8 (143.51–445.56)	0.51
HDL-C (mmol/L)	1.02 ± 0.36	1.05 ± 0.22	0.60
LDL-C (mmol/L)	2.05 ± 0.93	2.14 ± 0.89	0.60
Triglycerides (mmol/L)	1.26 (0.84–1.94)	1.42 (1.03–2.14)	0.04
Total cholesterol (mmol/L)	3.79 ± 1.41	4.01 ± 1.04	0.33
Prealbumin (g/L)	0.26 ± 0.10	0.33 ± 0.12	0.02
Ccr (ml/min)	6.67 ± 1.67	7.23 ± 1.73	0.08
TMAO (ng/mL)	82.52 ± 18.93	72.33 ± 20.19	< 0.01

Kt/V: urea clearance index; WBC: white blood cell; BUN: blood urea nitrogen; HDL-C: high-density lipoprotein cholesterol; LDL-C: low-density lipoprotein cholesterol; Ccr: endogenous creatinine clearance rate; TMAO: trimethylamine N-oxide.

### 3.2. Comparison of body composition between PEW and non-PEW groups in MHD patients

As shown in Table 2, in MHD patients, the PEW group exhibited lower levels of SLM, BMI, PBF, AMC, and PHA compared to the non-PEW group, while ECW/TBW ratio was higher in the PEW group than in the non-PEW group. These differences were statistically significant (*p* < 0.05). However, there were no statistically significant differences between the two groups in SMI.

**Table 2.** Comparison of body composition between MHD patients in PEW and non-PEW groups.

Variables	PEW group	non-PEW group	*P* value
	*N* = 73	*N* = 54	
BMI (kg/m^2^)	21.24 ± 2.95	24.30 ± 3.81	< 0.01
SLM (kg)	42.26 ± 9.72	46.95 ± 8.25	0.01
SMI (kg/m^2^)	7.23 ± 2.03	7.72 ± 1.28	0.10
PBF (%)	18.98 ± 9.31	22.73 ± 10.18	0.03
AMC (cm)	18.33 ± 2.00	19.30 ± 1.30	< 0.01
ECW/TBW	0.40 ± 0.01	0.39 ± 0.01	0.03
PHA (°)	4.38 ± 1.81	4.92 ± 0.97	0.04

BMI: body mass index; SLM: soft lean mass; SMI: skeletal muscle index; PBF: percent of body fat; AMC: arm muscle circumference; ECW/TBW: extracellular water/total body water; PHA: phase angle.

### 3.3. Analysis of factors influencing the development of PEW in MHD patients

After excluding the indicators used in the PEW diagnostic criteria, potential indicators influencing the occurrence of PEW were included as independent variables for binary logistic regression analysis, with the occurrence of PEW as the dependent variable. Binary logistic regression analysis indicated that Cr, Hb, TMAO, ECW/TBW ratio, and PBF were significantly associated with the occurrence of PEW in MHD patients (*p* < 0.05). Subsequently, after including age, sex, Cr, Hb, TMAO, ECW/TBW ratio and PBF as independent variables, multivariate logistic regression analysis was conducted. The results showed that after full adjustment, TMAO and ECW/TBW ratio were independent risk factors for PEW in MHD patients (*p* < 0.05), as shown in Table 3.

**Table 3.** logistic regression analysis of PEW occurrence in MHD patients.

Variables	Univariate regression		Multivariate regression	
	OR value (95%CI)	*P* value	OR value (95%CI)	*P* value
Cr	0.99 (0.98–0.99)	0.02		
Hb	0.95 (0.90–0.99)	0.04		
TMAO	1.65 (1.15–2.37)	0.01	1.61 (1.08 ∼ 2.40)	0.02
PBF	0.86 (0.77–0.96)	0.02		
ECW/TBW	1.07 (1.01–1.14)	0.04	1.05 (1.01 ∼ 1.13)	0.04

TMAO: trimethylamine N-oxide; PBF: percent of body fat; ECW/TBW: extracellular water/total body water.

### 3.4. Correlation analysis of plasma TMAO with demographic and dialysis-related data, laboratory parameters, and body composition in MHD patients with PEW

**Figure 1.**
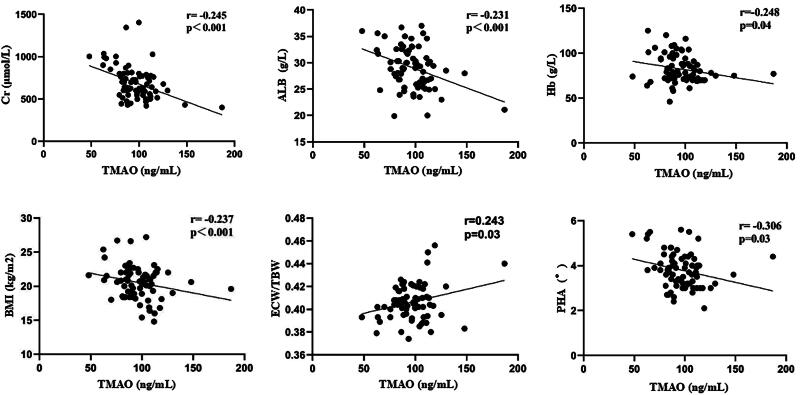
Correlation scatterplots of plasma TMAO with statistically significant laboratory parameters and body composition in MHD patients with PEW. TMAO: trimethylamine N-oxide; Cr: creatinine; ALB: albumin; Hb: hemoglobin; BMI: body mass index; ECW/TBW: extracellular water/total body water; PHA: phase angle.

Plasma TMAO levels correlated negatively with Cr (r = −0.245, *p* < 0.001), ALB (r = −0.231, *p* < 0.001), Hb (r = −0.248, *p* = 0.04), BMI (r = −0.237, *p* < 0.001), and PHA (r = −0.306, *p* = 0.03), and positively with ECW/TBW (*r* = 0.243, *p* = 0.03) in MHD patients with PEW, as shown in Figure 1.

### 3.5. The predictive efficacy of plasma TMAO for the occurrence of PEW in MHD patients

**Figure 2.**
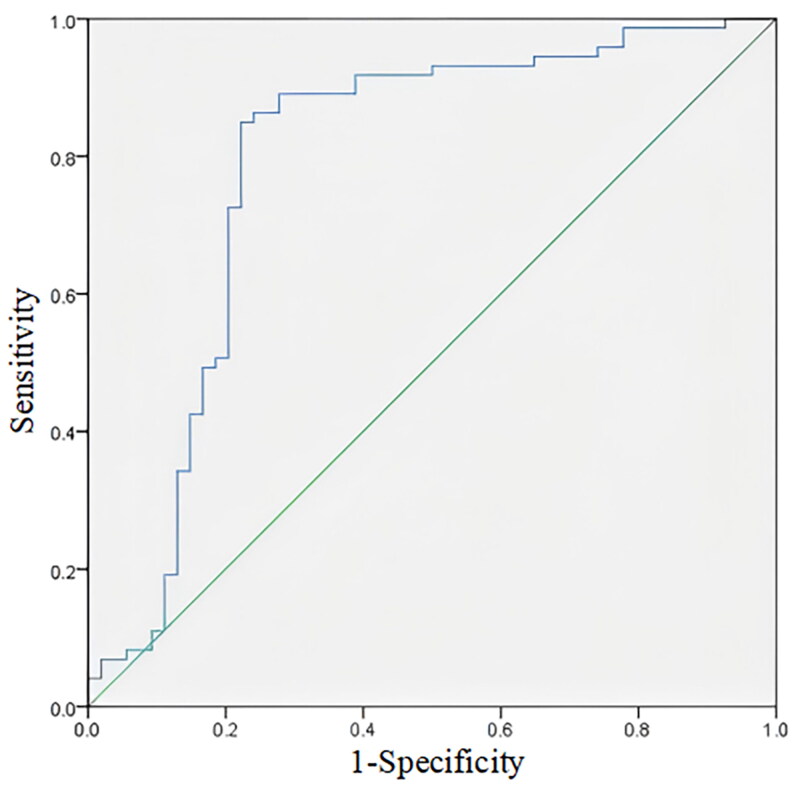
ROC curve of plasma TMAO for diagnosing PEW in MHD patients (AUC = 0.788).

The ROC curve shown in Figure 2 revealed that the optimal cutoff value of plasma TMAO was 81.31 ng/mL, with an area under the curve (AUC) of 0.788 (95% CI: 0.698–0.878, *p* < 0.001) for predicting PEW in MHD patients. The sensitivity was 84.9% and the specificity was 87.8% (*p* < 0.001).

## 4. Discussion

Currently, there is limited research on the relationship between TMAO, body composition, and PEW in MHD patients. This study indicated that circulating TMAO was an independent risk factor for PEW in MHD patients. This finding was similar to that of Chun Hu et al. [20]. However, a letter by Wenyun Wang et al. suggested that the study by Chun Hu et al. should account for several important factors influencing circulating TMAO, such as residual renal function (RRF), medications, inflammation, and diet, to avoid potential confounding effects [21].

This study assessed several key factors influencing circulating TMAO levels, thereby enhancing the credibility of the findings. First, RRF and hemodialysis: TMAO is an intestinal-derived microtoxin and cleared by the kidneys through a combination of glomerular filtration and tubular secretion [10]. Previous studies suggested that serum TMAO levels in MHD patients reflect the net effect of TMAO production and clearance, with the rate of TMAO removal by hemodialysis being comparable to that of creatinine [10,14]. Therefore, RRF and hemodialysis have a significant impact on TMAO clearance. There was no statistically significant difference in KT/V between the two patient groups (PEW and non-PEW). In addition, we collected fasting blood samples for TMAO measurement at least 36 h after a hemodialysis session (prior to the next hemodialysis session), in order to minimize the potential impact of a single dialysis session on TMAO levels. Clinically, endogenous creatinine clearance rate (Ccr) is commonly used to estimate residual renal function [22]. There was no statistically significant difference in creatinine clearance (CCr) between the PEW and non-PEW groups. Second, medications: Studies have shown that the use of medications such as metformin, sevelamer, iron supplements, proton pump inhibitors, antibiotics, aspirin, vitamins, and probiotics can affect plasma TMAO levels [23–25]. Aspirin, vitamin D3, and B vitamins can reduce plasma TMAO levels, whereas the use of metformin and sevelamer can increase circulating TMAO levels [23,25]. In this study, we excluded patients who had used these medications within the past month by reviewing their medical records and conducting interviews. This helped eliminate the influence of medications known to affect TMAO levels. Third, inflammation and infection: Research has shown that inflamed patients exhibit higher levels of TMAO [26]. In the PEW and non-PEW groups, CRP levels showed a statistically significant difference, whereas WBC counts did not. CRP is a common marker of inflammation, while elevated WBC counts are often associated with infections in MHD patients. PEW and inflammation interact with each other, which may account for the difference in CRP levels observed between the PEW and non-PEW groups [27]. Finally, diet: TMAO levels are influenced by diet. In this study, patient interviews were conducted, and exclusion criteria included the consumption of red meat, seafood, or eggs ≥5 times per week, as well as vegetarian or exclusively meat-based diets. This method mitigates the errors caused by dietary effects on TMAO levels to some extent.

In this study, ECW/TBW ratio was an independent risk factor for PEW in MHD patients. ECW/TBW is an indicator reflecting fluid balance in human body composition, with a higher ratio indicating a greater fluid overload burden [28]. This study found that the ECW/TBW ratio was significantly associated with the occurrence of PEW in MHD patients. The possible reasons considered are as follows: heavy volume loading may induce edema of the gastrointestinal mucosa, thereby causing decreased appetite, digestive dysfunction, and reduced protein absorption in patients, exacerbating their malnutrition status. Furthermore, mucosal edema compromises intestinal barrier function, disrupts gut microbiota ecology and distribution, leading to heightened systemic microinflammation [29].

Based on data from dialysis centers worldwide, the prevalence of PEW among MHD patients is higher compared to non-dialysis CKD patients [1]. In this study, the incidence of PEW among MHD patients was 57.48%. This study further investigated the predictive capability of plasma TMAO for PEW occurrence in MHD patients using ROC curve analysis. The area under the curve (AUC) was 0.788, suggesting that plasma TMAO appears to have predictive value for PEW occurrence in MHD patients. Currently, the pathophysiological mechanism between TMAO and PEW remains unclear. Research shows TMAO affects cholesterol and sterol metabolism [30]. We speculate that the pathophysiological mechanisms underlying the relationship between TMAO and PEW may be related to liver and biliary bile acid metabolism.

This study is a cross-sectional study, and its results may be influenced by Neyman bias. Our study did not include PEW patients who had either passed away or quickly recovered. The absence of such patients may lead to an overestimation or underestimation of the association between certain factors and PEW. Future prospective cohort studies are needed to more accurately clarify the relationship between TMAO and PEW.

## 5. Conclusion

This study shows plasma TMAO levels and certain body composition are associated with the occurrence of PEW in MHD patients. Plasma TMAO levels appear to serve as a potential predictive marker for the onset of PEW. These findings offer new insights into the identification of PEW in MHD patients and lay the groundwork for effective prevention and treatment strategies for PEW.

## Data Availability

All the data generated or analyzed during this study are included in this article. Further enquiries can be directed to the corresponding author.
